# Waves of inequality: income differences in intensive care due to Covid-19 in Sweden

**DOI:** 10.1093/eurpub/ckad094

**Published:** 2023-06-15

**Authors:** Karl Gauffin, Olof Östergren, Agneta Cederström

**Affiliations:** Department of Public Health Sciences, Stockholm University, Stockholm, Sweden; Department of Public Health Sciences, Stockholm University, Stockholm, Sweden; Aging Research Center (ARC), Karolinska Institutet, Solna, Sweden; Department of Public Health Sciences, Stockholm University, Stockholm, Sweden

## Abstract

**Background:**

Socioeconomically vulnerable groups were overall more likely to develop severe Covid-19, but specific conditions in terms of preparedness, knowledge and the properties of the virus itself changed during the course of the pandemic. Inequalities in Covid-19 may therefore shift over time. This study examines the relationship between income and intensive care (ICU) episodes due to Covid-19 in Sweden during three distinct waves.

**Methods:**

This study uses Swedish register data on the total adult population and estimates the relative risk (RR) of ICU episodes due to Covid-19 by income quartile for each month between March 2020 and May 2022, and for each wave, using Poisson regression analyses.

**Results:**

The first wave had modest income-related inequalities, while the second wave had a clear income gradient, with the lowest income quartile having an increased risk compared to the high-income group [RR: 1.55 (1.36–1.77)]. In the third wave, the overall need for ICU decreased, but RRs increased, particularly in the lowest income quartile [RR: 3.72 (3.50–3.96)]. Inequalities in the third wave were partly explained by differential vaccination coverage by income quartile, although substantial inequalities remained after adjustment for vaccination status [RR: 2.39 (2.20–2.59)].

**Conclusions:**

The study highlights the importance of considering the changing mechanisms that connect income and health during a novel pandemic. The finding that health inequalities increased as the aetiology of Covid-19 became better understood could be interpreted through the lens of adapted fundamental cause theory.

## Introduction

As more countries declare that they have entered the endemic phase of Covid-19, public health researchers will continue to study how the pandemic unfolded, and the measures that could have been taken to mitigate its impact on populations all over the world. Like with every new infectious disease, lessons can be learned from the past, while it is also true that each pandemic consists of unique combinations of characteristics in pathogens, hosts and environmental factors. For example, the pre-symptomatic transmission and high viral shedding in the early stage of a SARS-CoV-2 infection, along with a significant increase of international flight traffic from China over the past 15 years, may have contributed to the divergent epidemiological patterns when comparing the SARS outbreak of 2002–04 with the early stage of the recent pandemic.^1^ Some researchers even look further back by drawing parallels between Covid-19 and the 1918/19 influenza pandemic, identifying both similarities and differences.^2^ The overlap is not to be overemphasized, as past experiences may obscure important novelties in each new infectious disease. Particularities related to the transmission of Covid-19 were recognized early on, and phenomena like ‘cluster infections’ and ‘superspreading events’ became integrated into everyday terminology, shaping the mitigation strategies. Nevertheless, reflecting on the 3 years since the onset of the pandemic, there are perhaps more similarities to previous epidemics than initially expected. For example, similar to the influenza pandemics of the 20th century, Covid-19 has impacted the population in distinct seasonal waves, with infection, hospitalization and death rates decreasing substantially during the summer.^3^ This pattern has been particularly evident in European countries, where Covid-19-related death rates peaked at a 14-times higher level during the winter of 2020/21 compared to the preceding summer.^4^

### Preparations, conditions and reactions during three distinct waves

**Figure 1:**
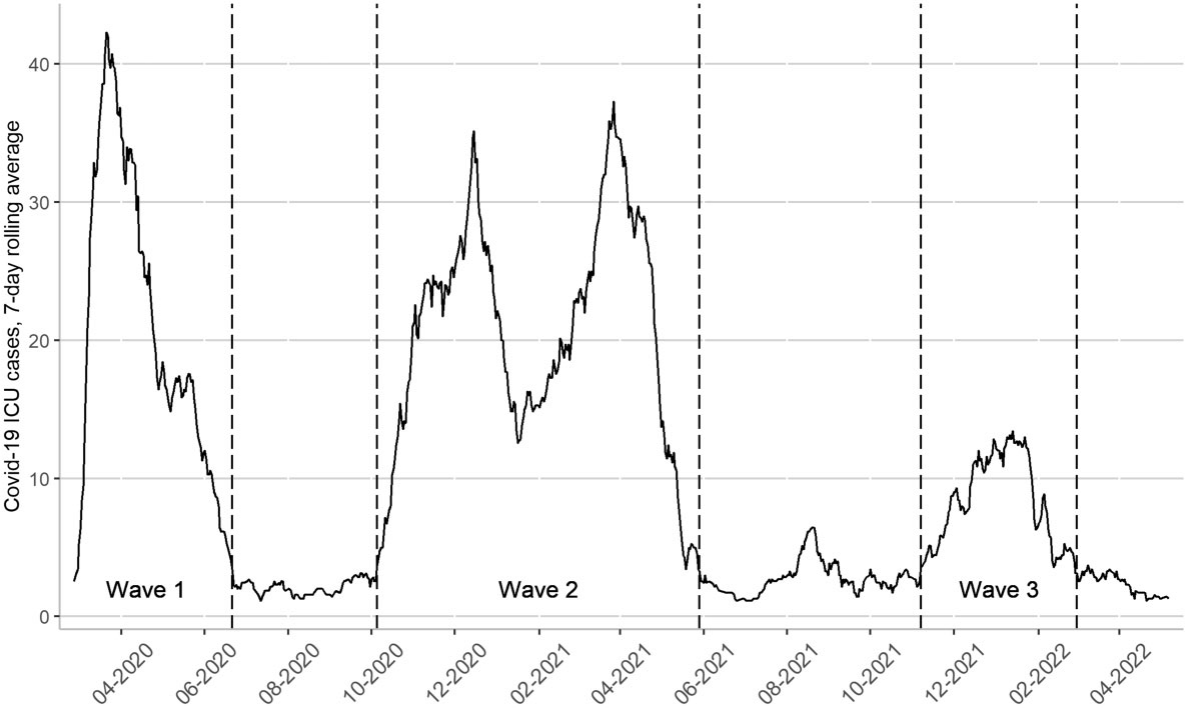
ICU due to Covid-19 in Sweden

The general pattern observed in Europe is also clear in individual countries. Sweden, a country hit hard by the first wave of the pandemic, experienced a sharp decrease in Covid-19 hospitalizations and deaths during the summer of 2020. The increasing infection rates following the summer led to a bimodal wave of Covid-19 in the winter of 2020/21, whereas the third wave following the summer of 2021 led to lower rates of intensive care (ICU) compared to most other European countries (see figure 1). These three waves can be differentiated by several characteristics that fundamentally influence how the disease affected the population.

Wave 1 caught the population, the healthcare system and political leaders off guard. On 8 April 2020, there were 122 deaths in Sweden with Covid-19 listed as the underlying cause of death, the highest number observed during the first wave. Given that the incubation period of the alpha variant was likely around 5 days^5^ and the time from diagnosis to death was estimated to be around 18 days,^6^ it is probable that the peak of infections occurred well before the first substantial population-wide interventions were implemented on 1 April 2020.^7^ Additionally, widespread testing was not available to the general public during the first wave,^8^ resulting in limited use of isolation and contact tracing. In comparison, although Wave 2 was also driven by rapidly increasing infection rates, the experiences gained during the spring diminished the sense of exceptionalism, and the population had to adapt to living with Covid-19. During this period, those who were able to had made significant adjustments to their daily lives. The healthcare system improved its ability to detect and treat cases, polymerase chain reaction testing became more widely accessible^8^ and the 60-day mortality rate of patients admitted to hospitals remained significantly lower compared to the first wave.^9^ These improvements, along with changes in social and health insurance rules, provided individuals with additional opportunities to mitigate the negative consequences of the pandemic. Wave 3 was characterized by the rapid spread of the more infectious, but less severe, omicron variant. This surely contributed to the relatively low ICU rates in the first months of 2022, despite the unprecedented number of infections.^10^ However, the primary protecting factor in this wave was the progressively increasing vaccination rate of the population.^11^

### Social inequalities during the pandemic

In addition to the epidemiological waves, another historical parallel can be drawn concerning social inequalities in health and illness. In a comparative review, Mamelund and Dimka^12^ present studies demonstrating that, similar to Covid-19, the 1918/19 influenza had a greater impact on the working class and disadvantaged groups compared to the more privileged population. Likewise, for Covid-19, there were early indications that in the initial phase of the pandemic, the virus spread more among those in higher socioeconomic position,^13^ partly due to greater international mobility and more extensive social contacts.^14^ Evidence suggests that travellers returning from the Alps, particularly Austria, were responsible for much of the initial introduction of the virus in Sweden,^15^ and regions with school winter breaks during the week when the European infection rates surged were hit especially hard in the first wave.^16^ On the other hand, public health agencies and the research community expressed concerns that the pandemic had the potential to exacerbate existing health inequalities, with vulnerable groups having fewer protective resources and experiencing a greater burden of medical risk factors.^17^ The pandemic has been described as a ‘syndemic’; referring to the interplay between infectious diseases and stress, poverty and other forms of structural disadvantage.^18^ The discussion surrounding the ‘unequal pandemic’ emerged early on, and studies confirm clear patterns of covid-19-related health inequalities in several countries.^19^ Swedish studies focussing on the first year of the pandemic reveal varying degrees of associations between socioeconomic disadvantage and covid-19, depending on the defined study period and specifics outcome (e.g. hospitalization, ICU or death).^20–22^

This study aims to contribute to the understanding of Covid-19-related health inequity by investigating income inequalities in ICU episodes due to Covid-19 between March 2020 and May 2022. By separating the three different waves in Sweden, we can analyze and discuss how the mechanisms that potentially produce socioeconomic inequalities in severe Covid-19 may have shifted over the course of the pandemic. As our understanding of the disease improves and more effective prevention and treatment measures emerge, individuals with greater resources are more likely to be able to utilize that knowledge to protect themselves. Therefore, we anticipate that the socioeconomic gradient in severe Covid-19 become more pronounced with subsequent waves.

## Methods

Swedish administrative population registers contain information on various socioeconomic conditions and healthcare use. These registers offer researchers in social epidemiology and health equity studies excellent individual-level data for longitudinal population studies. Each Swedish resident is assigned a personal identification number (PIN) at birth or upon public registration of residence for foreign-born individuals. The use of PINs enables record linking, which allows for the tracking of individuals from birth to death across different registers. For privacy reasons, the PINs are anonymized when used in research. The data used in this study have been obtained in collaboration with the Swedish Public Health Agency and the study has been approved by the Swedish Ethical Review Authority (D.Nr. 2021-05754-02).

### Study population, measures and outcomes

The study population was defined as the total population over the age of 20 residing in Sweden at the end of 2019. Information on birth year, sex and country of birth was retrieved from the Total Population Register. Family disposable income and size were obtained from the Longitudinal Integrated Database for Health Insurance and Labour Market Studies (LISA). Data on Covid-19-related ICU hospitalizations were collected from the national notifiable disease registry (SmiNet) and the Swedish Intensive Care Register, while information on Covid-19 vaccinations was obtained from the National Vaccination Register. The population was divided into four income quartiles based on family-level disposable income in 2019, as listed in the National Register of Income and Taxes. The income was equivalized by the square root of the number of family members and categorized as high, mid-high, mid-low and low.

In this study, we use ICU episodes as an indicator of severe cases of Covid-19, which offers several advantages over other potential indicators. ICU treatment for Covid-19 typically involves invasive and physically demanding forms of medical intervention, which are unlikely to be administered as a precautionary measure. While deaths due to Covid-19 are an alternative measure, the majority of deaths occurred among older individuals receiving at-home care or residing in nursing homes.^23^ ICU episodes, on the other hand, provide an indicator that is applicable across a broad age range and remained relatively stable throughout the study period. We defined the outcome as the first event of ICU due to Covid-19.

### Statistical analysis

We conducted Poisson regression analyses to estimate the relative risk (RR) of ICU episodes with Covid-19 by income quartile. The analysis was performed separately for each month between March 2020 and May 2022 and for each of the three distinct waves. The follow-up period was defined from 1 March 2020 until the first ICU hospitalization from Covid-19, death, or the end of follow-up on 1 June 2022. To examine how the income gradient evolved during the pandemic, we divided the follow-up period first by month, using the first day of each month as a breakpoint. We also divided the follow-up by waves, using the dates 5 July 2020 (end of Wave 1), 19 October 2020 to 12 June 2021 (Wave 2) and 21 November 2021 to 15 March 2022 (Wave 3) as breakpoints, as illustrated in figure 1. These dates were determined based on the 7-day rolling average of ICU hospitalizations due to Covid-19, and the breakpoints were set when the average fell below three cases per day.

The models were adjusted for age, sex and region of origin. We estimated the interaction between income and month as well as income and wave. Additionally, we examined the effect of differential vaccination rates by cutting the follow-up at the date of the first vaccination for each individual. Sensitivity analyses were conducted, including models without adjustment for region of origin, and a three-way interaction with sex. The log of the follow-up duration was included as an offset in the models, and confidence intervals were estimated using robust standard errors. All statistical analyses were performed using R version 4.2.1, with the Lexis functions in the Epi package utilized to cut the data.

## Results

During the study period, a total of 8751 persons received ICU due to Covid-19 in Sweden. Table 1 presents the total number of individuals, percentages, number of ICU cases with Covid-19, crude incidence rates and vaccination rates per income group.

**Table 1:** Number and incidence rates of ICU cases with Covid-19

Total	*N* (%)	ICU	ICU incidence rate^a^
	7 982 703 (100)	8751	49.4
**Income quartiles**			
High	1 998 799 (25.0)	1525	34.0
Mid-high	1 998 140 (25.0)	1937	43.3
Mid-low	1 995 849 (25.0)	2289	51.7
Low	1 989 915 (24.9)	3000	69.2

a Per 100 000 person-years.

**Figure 2:**
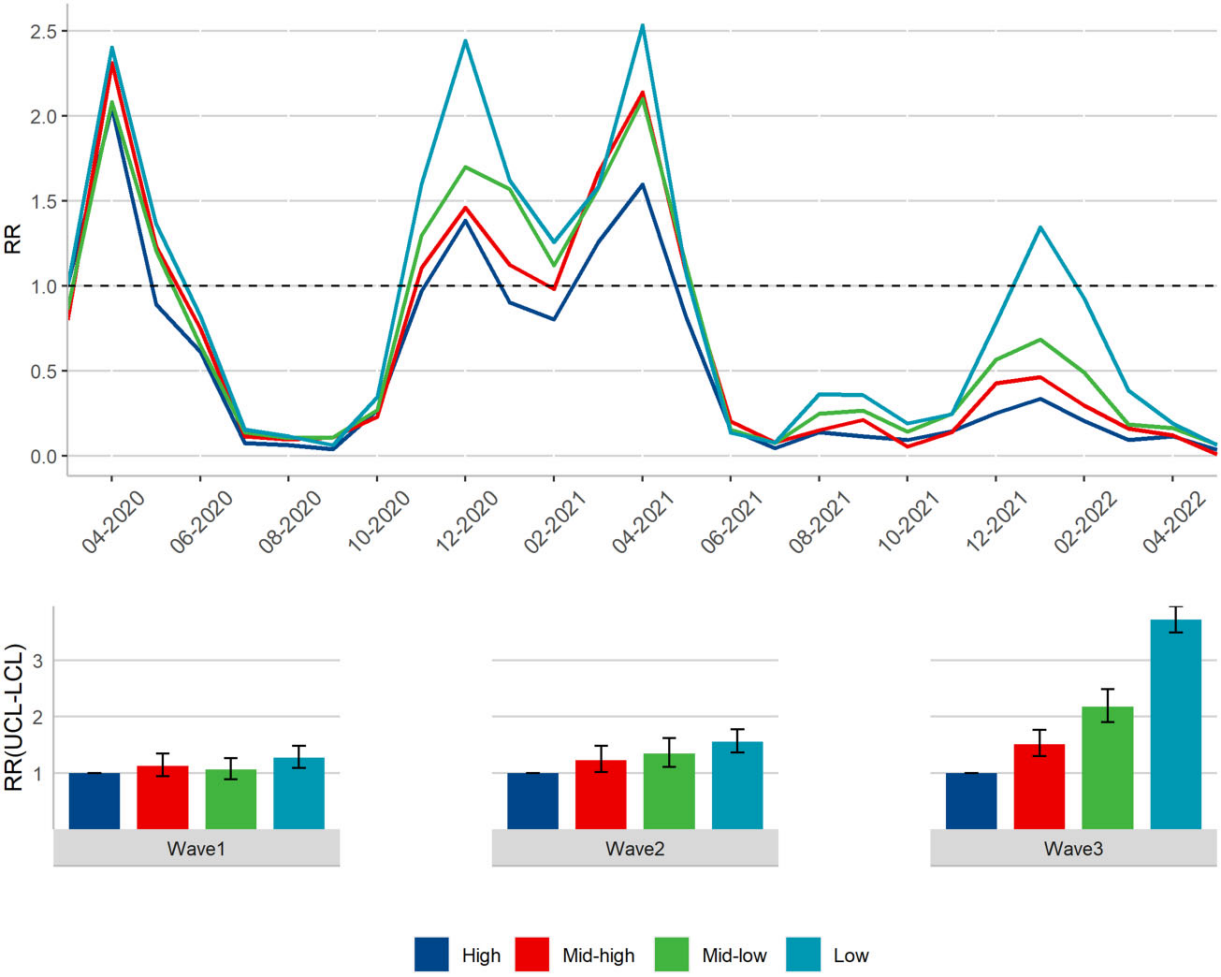
RRs for ICU per month and wave by income quartiles. Model includes age, sex and region of origin

In figure 2, the top panel shows the RRs for ICU per month by income quartile with those in the highest income quartile in March 2020 being the point of reference. The bottom panel shows the RRs per wave, now with the highest income quartile for each separate wave being the reference. The two graphs show complementary information, with the line graph depicting the complete development for the income quartiles per month while the bar graph shows how the RRs compare in each wave. In March and April 2020, there was no significant income gradient in the RRs for ICU, although by May 2020, the high-income quartile had a lower risk [RR: 0.89 (0.67–1.18)] compared to all other income quartiles, particularly the low-income quartile [RR: 1.36 (0.77–2.42)]. This disparity is reflected in the RRs within Wave 1, where only the low-income quartile demonstrated elevated risks for ICU [RR: 1.27 (1.08–1.48)]. After a summer characterized by low hospitalization rates and non-significant social differentiation, Wave 2 revealed a clear income-based gradient in the risk for ICU, with elevated risks for the mid-high [RR: 1.22 (1.02–1.48)], mid-low [RR: 1.34 (1.11–1.62)] and low [RR: 1.55 (1.36–1.77)] income quartiles compared to the high-income quartile. These inequalities were intensified in the third wave, although the overall risk for ICU decreased. Within Wave 3, however, the RR in the low-income quartile was substantial [RR = 3.72 (3.50–3.96)]. Tables providing the numerical estimates from which the graphs were derived from are available as Supplementary material.

**Figure 3:**
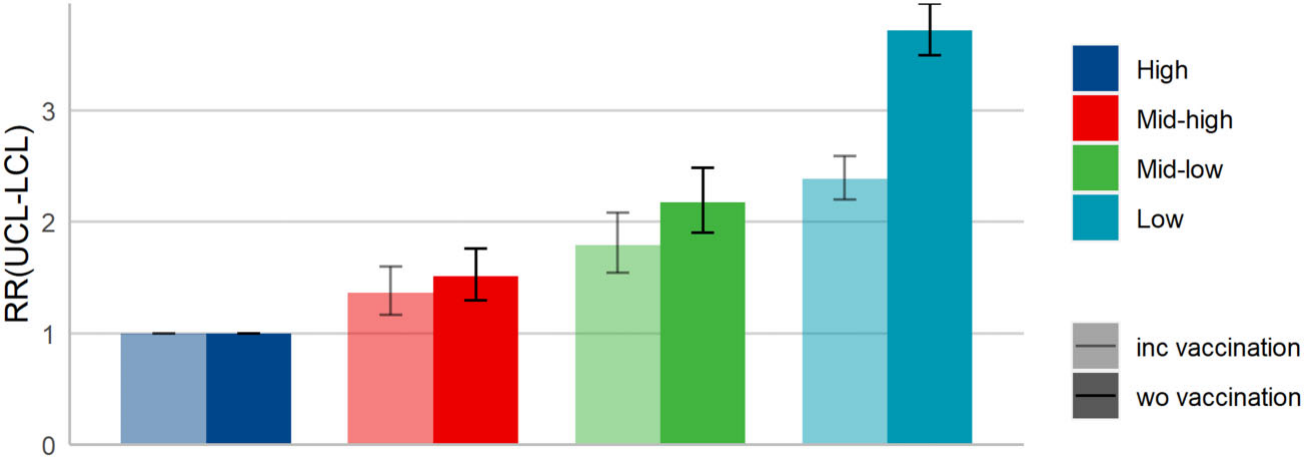
Income-related RRs in Wave 3 for ICU comparing models including and without vaccination status. Model includes age, sex and region of origin

Of particular interest is the extent to which disparities in vaccine uptake can account for the excess risk observed in the third wave, when all adult individuals in Sweden had been offered vaccinations. Figure 3 shows the income-related RRs in Wave 3, incorporating models that include vaccination status. Income differences in vaccination status explain a proportion of the elevated RR, particularly in the low-income group [RR: 2.39 (2.20–2.59)], although a considerable excess risk remains in all groups compared to the high-income reference quartile.

### Sensitivity analyses

The Supplementary figure S1 illustrates the development of RRs per income quartile per month, with the regression model adjusted only for age and sex and not region of origin. This was to ensure that the observed patterns were not an artefact of adjusting for migrant groups known to be vulnerable to the more serious consequences of Covid-19, such as mortality. The graph shows the same pattern of increasing inequalities by income quartile as the pandemic progressed, except that in the first month, the low-income quartile had a significantly higher risk. Moreover, the inequalities were actually larger in the second and third waves compared to when adjusting for region of origin.

The Supplementary figure S2 shows the moderating effect of sex on the relationship between income quartiles and months by including a three-way interaction term. The analysis reveals a significant moderating effect, indicating that for women, unlike men, there was already a gradient by income in ICU with Covid-19 in the first months. However, the disparities were exacerbated in the second and third waves for both sexes.

## Discussion

In this study investigating income inequalities in ICU for Covid-19, we observed a strong temporal relationship, with increasing disparities between income quartiles as the pandemic progressed. Our findings differ from some earlier studies that reported pronounced income gradients already in the first wave.^20^^,^^21^ However, these studies vary in terms of outcome, income measurement, other covariates and study population.

For public health researchers, the pandemic has prompted a re-evaluation of health inequality theories, which can inform our understanding of the epidemiology of Covid-19 in the three pandemic waves. The initial phase, characterized by modest or non-existent income inequalities, may be explained by previous findings on the socially stratified diffusion of novel diseases where knowledge about appropriate protection is lacking.^24^ The distribution of a disease in this stage may be somewhat random or influenced by competing socioeconomic patterns in risk exposure. For instance, in the case of Covid-19 in Sweden, initial exposure was greater in the upper middle class due to trips to alpine skiing resorts, while risk exposure was linked to socioeconomic disadvantage once the virus had arrived in the country.^7^ Similar competing socioeconomic patterns in risk exposure have been observed in diseases like skin cancer, with more privileged socioeconomic groups in northern Europe being more likely to travel abroad but less likely to work outdoors.^25^ Ambiguous trends have also been observed in relation to breast cancer, where higher socioeconomic position is associated with higher incidence (partly explained by reproductive factors), but lower case fatality.^26^ The absence of a socioeconomic gradient found in our study puts previous studies on Covid-19-related health inequalities during the first pandemic wave in Sweden into perspective. By analyzing the monthly changes in socioeconomic risk differences, the study provides a detailed picture of how Covid-19, within a matter of weeks, evolved from a novel and unknown condition to an established disease with well-investigated risk factors.

According to fundamental cause theory, health inequalities emerge as soon as disease aetiology and suitable protection measures are identified, because flexible resources such as money, knowledge, power and networks can be targeted to ensure better health outcomes in the more privileged population.^27^ Moreover, previous research has shown stronger social gradients in mortality from causes amenable to healthcare or behavioural change, relative to other causes.^28^ The second wave of the pandemic could be described as a ‘home office wave’ due to the increasing number of employees working from home in autumn and winter 2020/21. However, it is important to acknowledge that not everyone had the option to protect themselves through remote work. Disparities in rates of different occupational groups working remotely highlight the gap between public health recommendations and reality. For example, while around 66% of Swedish managers and employees with advanced levels of higher education reported working from home in the first quarter of 2021, the corresponding rate among workers in service, care and sales occupations was only about 6%. This demonstrates that for many people, non-exposure to high-risk-environments was not an option.^29^ Applying the criteria of fundamental cause theory to the relationship between socioeconomic position and Covid-19 implies exploring the multidimensionality and flexibility of income in minimizing the risk for severe Covid-19. Here, income serves a fundamental cause of Covid-19-related health inequalities by (i) influencing multiple diseases that interact adversely with a Covid-19 infection, making the disease more severe, (ii) affecting disease outcomes through multiple risk factors, (iii) providing access to resources that assist in minimizing virus exposure or disease vulnerability and (iv) its flexibility in reproducing the association with the disease via replaced mechanisms.

The flexibility of socioeconomic resources in protecting against Covid-19 became evident with the roll-out of the game-changing vaccines. The relatively low rates of ICU during the third wave can largely be attributed to the effective immunization efforts that began in Sweden in January 2021. However, it soon became apparent that Covid-19-related inequalities would persist, partly due to socioeconomic patterns in vaccine uptake, as confirmed in this study. While the relative income gradient was more pronounced in the third wave, the largest number of excess cases of severe Covid-19 by income was observed during the second wave. This development is similar to the general pattern of the cigarette epidemic where social gradients in smoking tend to emerge in the later stages as it becomes less common in the total population.^30^ Although disparities in vaccination rates contribute to the inequalities seen in the third wave, they cannot completely account for them. It is likely that there are other underlying health characteristics that are the main drivers of this residual inequality, as exposure to the virus was not differentiated when the country had opened up almost completely. The limitation of not having access to previous health records of the study population could be addressed in future research. Additionally, a more differentiated socioeconomic measure, including education and occupation, could potentially provide a more nuanced understanding of the relationship between social inequality and Covid-19-related health disparities. Different indicators of socioeconomic position, although correlated, capture different aspects of socioeconomic position and may be associated with Covid-19 morbidity through distinct mechanisms. Occupation can reflect exposure to the virus and the ability to work remotely, education may correlate with information-seeking and decision-making abilities, which were particularly relevant during the pandemic with the abundance of new information and misinformation, while income represents available resources that can be utilized to protect individuals. When using a single indicator, as done in this study, it serves as a proxy measure for the other two.^31^ Future studies aiming to further elucidate the mechanisms underlying the socioeconomic inequalities demonstrated in this study should incorporate multiple indicators of socioeconomic position. In conclusion, as knowledge about prevention and treatment strategies become more established, overall incidence rates decline, suggesting that disadvantaged groups may be particularly vulnerable during an intermediate phase. This highlights the importance of strengthening public health efforts during this critical window. Implementing combined measures to reduce adverse exposures, vulnerabilities and social consequences of disease in disadvantaged groups, along with evidence-based strategies to increase vaccination rates in the general population, are crucial for promoting health equity during a novel pandemic.

## Supplementary Material

ckad094_Supplementary_Data

## Data Availability

The study was done using data from the Swedish population registers. The Public Access to Information and Secrecy Act in Sweden prohibits the authors from making individual-level data publicly available. Researchers interested in replicating this work can apply for individual-level data from Statistics Sweden and the National Board of Health and Welfare.
