# Supplementary material for: Waves of inequality: income differences in intensive care due to Covid-19 in Sweden
Source: Eur J Public Health. 2023 Jun 15;33(4):574–9. doi: 10.1093/eurpub/ckad094 (PMC10393505; doi:10.1093/eurpub/ckad094)
Supplement: ckad094_Supplementary_Data [file ckad094_supplementary_data.zip › ckad094_Supplementary_Data/ejph-2023-03-om-0139-File006.docx]

## Supplementary table 1: Relative risks for intensive care per month and wave by income quartiles

|  | High income | Mid–high income | Mid–low income | Low income |
| --- | --- | --- | --- | --- |
| 20-mar | 1 (ref) | 0.80 (0.57–1.12) | 0.86 (0.64–1.16) | 0.99 (0.74–1.33) |
| 20-apr | 2.05 (1.63–2.57) | 2.31 (1.18–4.52) | 2.08 (1.15–3.78) | 2.40 (1.33–4.34) |
| 20-may | 0.89 (0.67–1.18) | 1.23 (0.61–2.49) | 1.21 (0.67–2.17) | 1.36 (0.77–2.42) |
| 20-jun | 0.61 (0.48–0.79) | 0.75 (0.38–1.48) | 0.65 (0.34–1.23) | 0.82 (0.46–1.46) |
| 20-jul | 0.07 (0.03–0.17) | 0.11 (0.05–0.27) | 0.14 (0.06–0.32) | 0.15 (0.07–0.32) |
| 20-aug | 0.06 (0.03–0.15) | 0.10 (0.04–0.24) | 0.11 (0.04–0.27) | 0.12 (0.05–0.25) |
| 20-sep | 0.04 (0.02–0.08) | 0.11 (0.04–0.27) | 0.11 (0.04–0.29) | 0.06 (0.02–0.17) |
| 20-oct | 0.27 (0.18–0.41) | 0.23 (0.11–0.50) | 0.27 (0.12–0.60) | 0.35 (0.18–0.65) |
| 20-nov | 0.97 (0.72–1.30) | 1.11 (0.54–2.25) | 1.30 (0.71–2.36) | 1.60 (0.91–2.83) |
| 20-dec | 1.39 (1.04–1.85) | 1.46 (0.75–2.83) | 1.70 (0.96–3.02) | 2.44 (1.40–4.27) |
| 21-jan | 0.90 (0.68–1.20) | 1.13 (0.57–2.24) | 1.57 (0.87–2.82) | 1.62 (0.91–2.87) |
| 21-feb | 0.80 (0.58–1.11) | 0.98 (0.51–1.90) | 1.12 (0.60–2.10) | 1.26 (0.71–2.23) |
| 21-mar | 1.26 (0.97–1.63) | 1.67 (0.86–3.22) | 1.58 (0.88–2.81) | 1.58 (0.90–2.76) |
| 21-apr | 1.60 (1.26–2.03) | 2.14 (1.13–4.06) | 2.10 (1.17–3.77) | 2.53 (1.44–4.47) |
| 21-may | 0.81 (0.58–1.14) | 1.07 (0.50–2.26) | 1.12 (0.57–2.17) | 1.06 (0.58–1.95) |
| 21-jun | 0.15 (0.06–0.39) | 0.20 (0.08–0.52) | 0.15 (0.06–0.39) | 0.14 (0.06–0.31) |
| 21-jul | 0.05 (0.02–0.14) | 0.08 (0.03–0.25) | 0.07 (0.03–0.21) | 0.08 (0.03–0.21) |
| 21-aug | 0.14 (0.07–0.28) | 0.15 (0.06–0.42) | 0.25 (0.11–0.57) | 0.36 (0.17–0.75) |
| 21-sep | 0.12 (0.06–0.23) | 0.21 (0.09–0.53) | 0.27 (0.12–0.58) | 0.36 (0.18–0.72) |
| 21-oct | 0.09 (0.04–0.20) | 0.05 (0.01–0.20) | 0.14 (0.06–0.32) | 0.19 (0.08–0.43) |
| 21-nov | 0.14 (0.08–0.25) | 0.14 (0.05–0.36) | 0.25 (0.10–0.60) | 0.25 (0.12–0.51) |
| 21-dec | 0.25 (0.15–0.41) | 0.43 (0.19–0.99) | 0.57 (0.28–1.17) | 0.79 (0.41–1.52) |
| 22-jan | 0.34 (0.22–0.52) | 0.46 (0.22–0.98) | 0.69 (0.36–1.31) | 1.35 (0.74–2.44) |
| 22-feb | 0.21 (0.13–0.34) | 0.30 (0.13–0.66) | 0.49 (0.25–0.98) | 0.93 (0.50–1.73) |
| 22-mar | 0.09 (0.05–0.16) | 0.16 (0.06–0.40) | 0.18 (0.09–0.39) | 0.38 (0.19–0.79) |
| 22-apr | 0.12 (0.07–0.20) | 0.12 (0.05–0.32) | 0.16 (0.07–0.37) | 0.19 (0.08–0.45) |
| 22-may | 0.04 (0.02–0.08) | 0.01 (0.00–0.08) | 0.07 (0.02–0.19) | 0.06 (0.02–0.19) |
