# Supplementary material for: Waves of inequality: income differences in intensive care due to Covid-19 in Sweden
Source: Eur J Public Health. 2023 Jun 15;33(4):574–9. doi: 10.1093/eurpub/ckad094 (PMC10393505; doi:10.1093/eurpub/ckad094)
Supplement: ckad094_Supplementary_Data [file ckad094_supplementary_data.zip › ckad094_Supplementary_Data/ejph-2023-03-om-0139-File007.docx]

## Supplementary table 2: Relative risks for intensive care per income quartile for each wave

|  | High income | Mid–high income | Mid–low income | Low income |
| --- | --- | --- | --- | --- |
| Wave 1 | 1 (ref) | 1.12 (0.94–1.34) | 1.06 (0.89–1.27) | 1.27 (1.09–1.48) |
| Wave 2 | 1 (ref) | 1.23 (1.02–1.48) | 1.34 (1.11–1.62) | 1.55 (1.36–1.78) |
| Wave 3 | 1 (ref) | 1.51 (1.29–1.76) | 2.18 (1.90–2.48) | 3.72 (3.50–3.96) |
