# Supplementary figures and images for: Waves of inequality: income differences in intensive care due to Covid-19 in Sweden
Source: Eur J Public Health. 2023 Jun 15;33(4):574–9. doi: 10.1093/eurpub/ckad094 (PMC10393505; doi:10.1093/eurpub/ckad094)

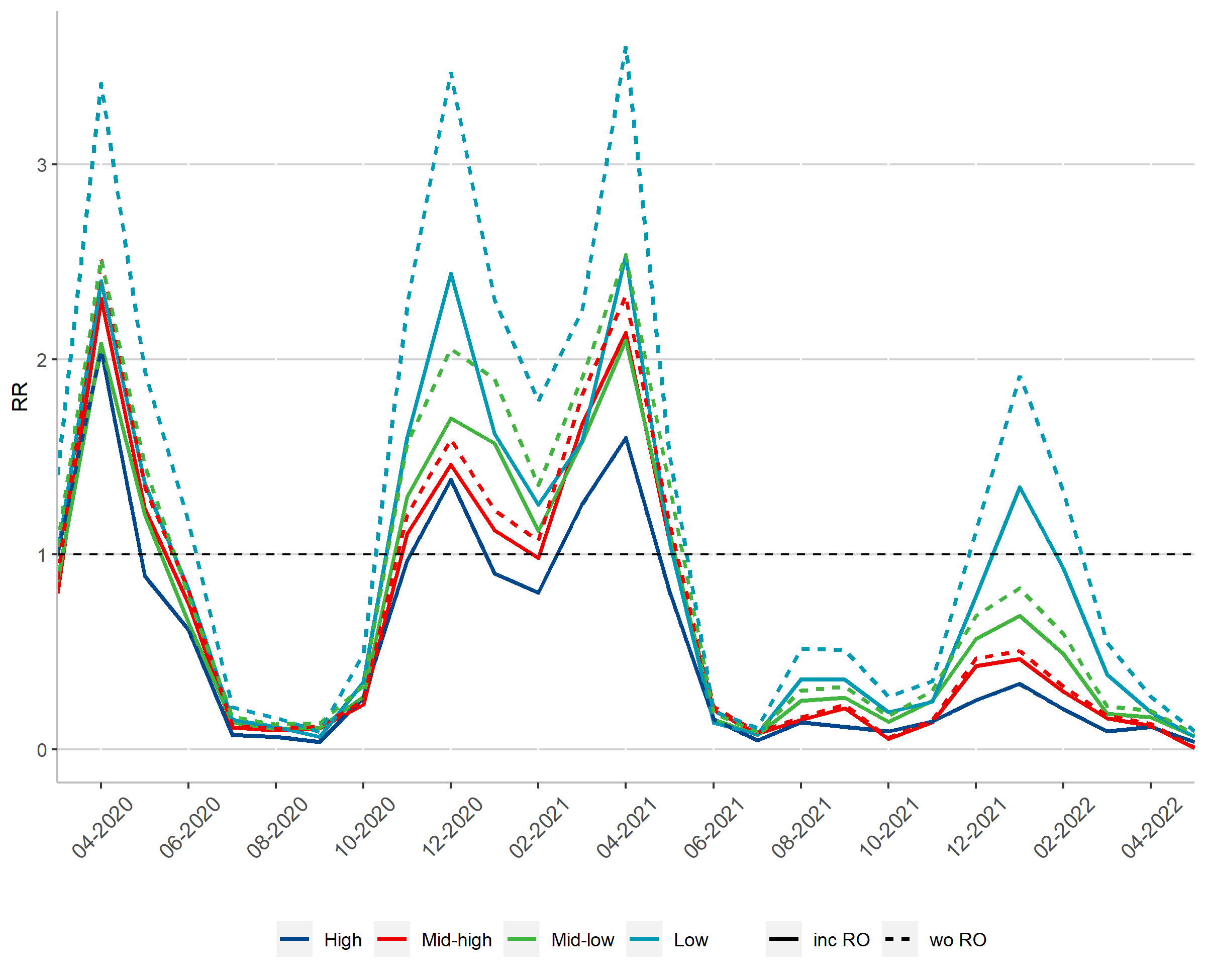

Supplement: ckad094_Supplementary_Data [file ckad094_supplementary_data.zip › ckad094_Supplementary_Data/ejph-2023-03-om-0139-File008.tiff]

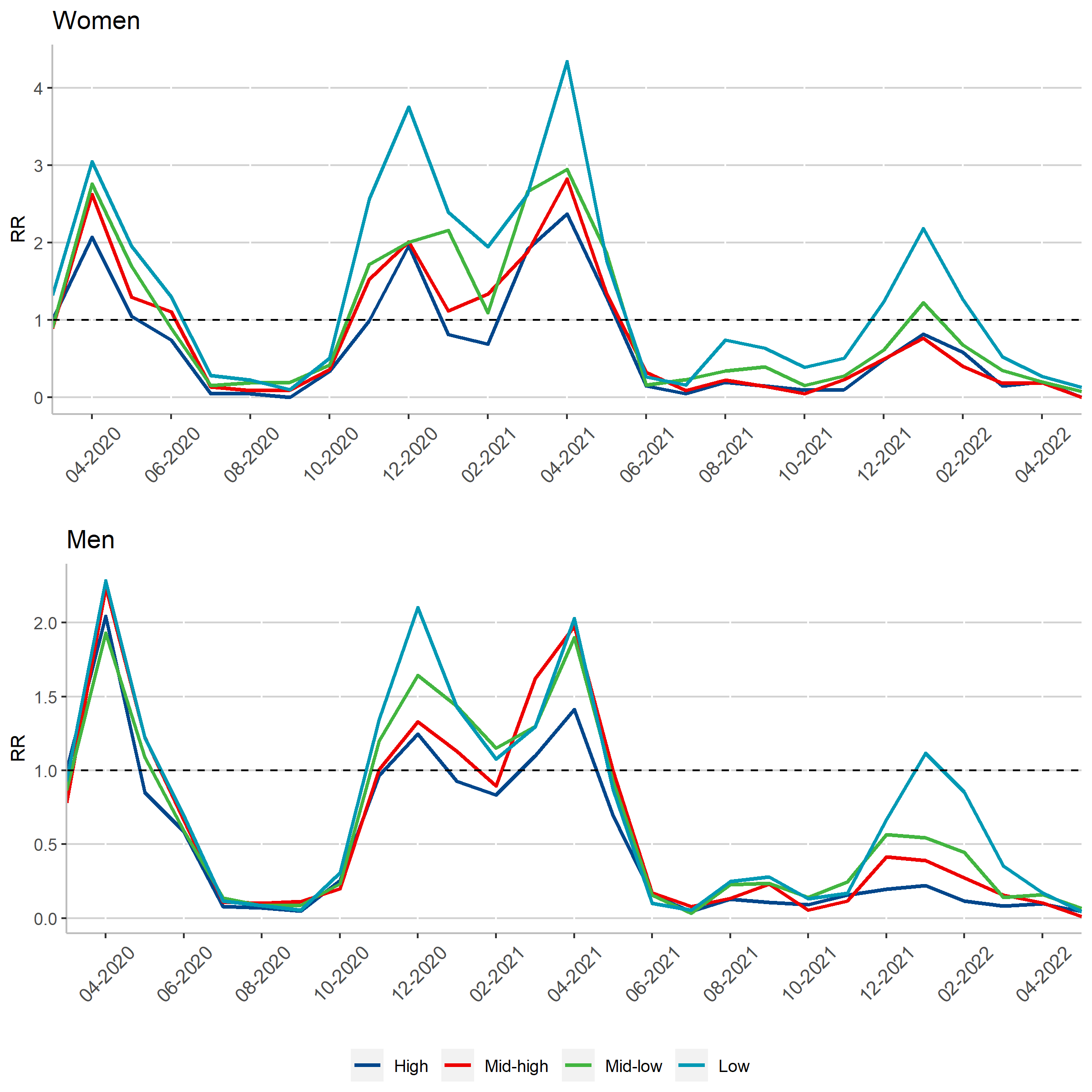

Supplement: ckad094_Supplementary_Data [file ckad094_supplementary_data.zip › ckad094_Supplementary_Data/ejph-2023-03-om-0139-File009.tiff]
